# The Dynamic Gut Microbiota: Monitoring Alterations During Lung Cancer Progression for Diagnosis and Precision Medicine

**DOI:** 10.3390/ijms27041905

**Published:** 2026-02-16

**Authors:** Xiao Xiao, Yi Wang, Tongxin Yin, Qiankun Wang, Yuting Feng, Huihao Ren, Jiaoyuan Li, Liming Cheng

**Affiliations:** Department of Laboratory Medicine, Tongji Hospital, Tongji Medical College, Huazhong University of Science and Technology, Wuhan 430030, China; xxiaoooo1009@163.com (X.X.); wangyi_200006@163.com (Y.W.); qiuqiu12462@163.com (T.Y.); qiankunwang.hust@foxmail.com (Q.W.); fytdoctor@163.com (Y.F.); rhhpolaris@163.com (H.R.)

**Keywords:** gut microbiota, metabolites, gut–lung axis, lung cancer

## Abstract

The gut microbiota, the body’s richest microbial ecosystem, is essential for maintaining gut function and immune balance. Additionally, microbial-derived metabolites are linked to the onset and progression of various diseases. There is a potential bidirectional gut–lung axis by which the gut and lungs can communicate with each other mediated by microbiota, immune responses, and metabolic products, and thus affect lung cancer occurrence. As the pathological progression of lung cancer advances and treatment methods are optimized, there is a concurrent and continuous alteration in the gut microbiota and its metabolites in lung cancer patients. This review highlights that the composition and structure of the gut microbiota in lung cancer patients undergo dynamic alterations, which are intricately linked to the pathological progression of the disease and the implementation of therapeutic interventions. Longitudinal monitoring of this system may offer unprecedented insights into the early diagnosis, precise treatment, and prognostic evaluation of lung cancer.

## 1. Introduction

Lung cancer is the most commonly diagnosed cancer worldwide, with estimated 2.5 million new cases occurred globally in 2022. The mortality of lung cancer account for 18.7% of all cancer deaths, making it a major cause of cancer-related mortality [1]. Non-small cell lung cancer (NSCLC) is the leading type of lung cancer, accounting for 85% of all lung cancer patients. While there have been considerable improvements in the diagnosis and treatment of lung cancer in recent years, many challenges still remain, including low screening accuracy, poor prognosis and survival for patients, and high risk of metastasis [2]. Therefore, lung cancer remains a severe public health issue, especially in less developed countries.

Risk factors for lung cancer include smoking, air pollution, genetic factors, poor diet and working exposures [3,4]. These factors do not directly cause cancer but trigger a complex process involving genetic mutations, epigenetic changes, chronic inflammation, and tumor microenvironment remodeling, all regulated by various interactions [5]. While the above factors are well-established risk factors for lung cancer, evidence now supports that gut microbiota have the potential to affect the pathological progression of lung cancer along the gut–lung axis via the circulatory system [6]. The gut microbiota consists of different microorganisms that colonize the human gastrointestinal tract, playing a significant role in shaping normal gut function, forming the gut barrier, and regulating host immunity [7]. Increasing evidence suggests that gut microbiota can form a certain crosstalk with the lungs, creating a “gut–lung axis”, which suggests that the gut microbiota and the lungs are interconnected through this axis, thereby exerting mutual influence on one another [8,9]. Gut microbiota and its metabolites have been discovered to be closely associated with the occurrence of lung cancer according to advanced research of genomics and metabolomics. The presently recognized microbial metabolites encompass short-chain fatty acids (SCFAs), tryptophan and its derivatives, polyamines, and secondary bile acids, among others [6]. The subsequent sections further elucidate the pivotal roles of gut microbiota and these metabolites in the development of lung cancer.

There is growing proof that lung cancer might influence the abundance of gut microbiota, and gut microbiota could affect the pathological changes in lung cancer [10]. Variations in gut microbiota composition have been observed in lung cancer patients across various stages of the disease and throughout different treatment processes, suggesting the presence of a discernible pattern [11,12]. These alterations in gut microbiota may partially reflect the pathological progression of lung cancer and the effectiveness of therapeutic interventions. Consequently, tracking the succession of the “dynamic gut microbiota” holds potential for aiding in the diagnosis of lung cancer and assessing treatment efficacy. In contrast to prior reviews that have predominantly regarded gut microbiota as static biomarkers, this review underscores the dynamic evolutionary patterns throughout the progression of lung cancer. It further discusses the distinctive value of this perspective in elucidating underlying mechanisms and informing clinical practice.

In this review, we mainly discuss the following aspects: (1) how the “gut–lung axis”, mediated by gut microbiota and its metabolites, interacts with lung cancer; (2) how the successional “dynamic gut microbiota” predicts the pathological progression and therapeutic efficacy of lung cancer; and (3) how some promising gut microbiota and metabolites provide strategies for adjuvant diagnosis and personalized treatment of lung cancer. Precise targeting of the interactions between gut microbiota and its metabolites with lung cancer may provide new insights for diagnosing and treating malignant tumors.

## 2. The Gut–Lung Axis in Lung Cancer

Despite the considerable distance between the gut and lungs, they communicate through microbial and immune functions, interconnected via the lymphatic and circulatory systems, forming the “gut–lung axis” for bidirectional regulation [13]. The gut microbiota affect the pathological progression of lung diseases by regulating immune responses and secreting metabolites. At the same time, lung inflammation can also lead to dysbiosis of the gut microbiota, potentially triggering intestinal diseases [14].

The gut microbiota significantly impacts the host immune system both locally and systemically. Locally, immune responses are triggered by pattern recognition receptors on intestinal cells detecting pathogen-associated molecular patterns like lipopolysaccharide and flagellin of gut microbiota. Additionally, microbial metabolites or microbes can activate dendritic cells, which move to mesenteric lymph nodes to activate naive T cells. These T cells differentiate into effector T cells, regulatory T cells, or T helper 17 cells (Th17 cells), which then migrate to the intestinal mucosa or enter the bloodstream, thereby systemically shaping the host’s immune response [15,16]. This systemic immunomodulatory capacity underpins the crosstalk within the gut–lung axis. A diverse gut microbiota supports the development of T helper 1 cells and M1 macrophages and enhances T cell activity, while increasing programmed cell death protein 1 (PD-1) expression on lymphocytes and fostering antitumor immunity [17,18]. Microbial metabolites like SCFAs further boost this effect by enhancing CD8+ T cell memory [19]. Clinically, in patients with lung cancer receiving anti-PD-1 therapy, individuals with higher gut microbial diversity show higher levels of memory CD8+ T cell and distinct natural killer cell (NK cell) subsets in their blood [20]. Another study showed that segmented filamentous bacteria can induce the migration of intestinal Th17 cells to the lungs through the gut–lung axis, enhancing antitumor effects by boosting IL-17A and IFN-γ production by CD4+ T cells, CD8+ T cells, and NK cells [21]. The gut microbiota modulates a complex immune system that maintains homeostasis and boosts antitumor immunity, making it a pivotal target for improving lung cancer treatment responses.

**Figure 1:**
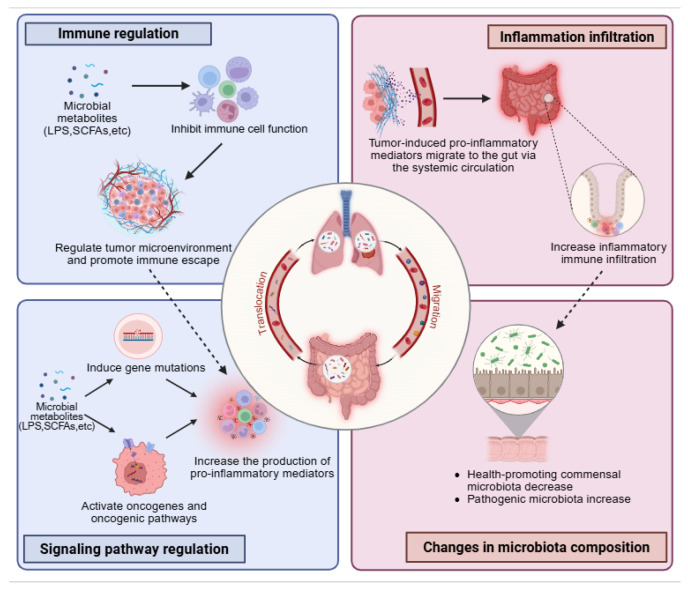
The mechanism of gut–lung axis interaction in lung cancer. The secretions of the gut microbiota (including their components and metabolites) can reach the lungs through the circulatory system, and a small amount of gut microbiota can also translocate to the lungs. They regulate the tumor microenvironment by suppressing the function of immune cells, thereby promoting immune escape. Moreover, these metabolites can directly induce gene mutations or activate oncogenes or oncogenic pathways. These pathways can all lead to the release of pro-inflammatory mediators. Conversely, pro-inflammatory factors from lung tumors can migrate to the gut, causing inflammation that reduces beneficial commensal microbiota and increases pathogenic microbiota. Created in BioRender. Xiao, X. (2026) https://BioRender.com/7zgoib4.

To date, the mechanisms by which gut microbiota influence lung disease development are still mostly unclear, but numerous studies have demonstrated the microbiome’s significant role in this process. It is considered that gut microbial components (such as peptidoglycan and lipopolysaccharides) and gut microbiota metabolites enter systemic circulation through the blood and lymphatic systems and are subsequently involved in the pathogenesis of lung cancer in three ways: (1) directly inducing gene mutations, (2) triggering oncogenes and oncogenic pathways, and (3) impairing immune cell function to regulate tumor microenvironment and promote immune escape. These three mechanisms can lead to chronic inflammation and increase the production of pro-inflammatory cytokines, chemokines, etc., which are crucial in the initiation and development of lung cancer [22,23]. Meanwhile, pro-inflammatory factors induced by lung tumors can migrate to the gut through systemic circulation, causing inflammatory infiltration in the gut. Long-term inflammatory infiltration can lead to dysbiosis (Figure 1) [13]. The crosstalk between the gut–lung axis in diseases is mainly reflected in the pathological progression of lung diseases altering the composition of gut microbiota, and changes in the gut microbiota can also affect lung disease progression.

There is a significant difference in the gut microbiota of lung cancer patients compared to healthy individuals [24]. Lung cancer patients have reduced gut microbiota diversity, with more pathogenic genera such as *Enterobacteriaceae*, *Streptococcus*, and *Prevotella*, and fewer beneficial genera such as *Faecalibacterium* and *Bifidobacterium* [25]. Another study found significant dysbiosis of butyrate-producing bacteria in the gut of NSCLC patients, including *Prevotella copri* (*P. copri*), *Fusobacterium nucleatum*, *Clostridium cluster I*, and *Ruminococcus* spp. [26]. Nevertheless, these investigations have thus far been confined to purely analytical approaches, lacking subsequent experimental validation. It is imperative that further experimental studies be conducted to substantiate the potential of these gut microbial signatures as biomarkers for lung cancer.

These evidences suggest that there is some connection between the lungs and the gut. Lung cancer patients may experience dysbiosis of the gut microbiota, but the underlying mechanisms remain unclear and require further research to elucidate. In summary, the changes in gut microbiota characteristics observed in lung cancer patients suggest that gut microbiota are crucial for the pathogenesis of lung cancer.

## 3. Monitoring Dynamic Gut Microbiota to Predict Pathological Progression and Therapy Efficacy in Lung Cancer Patients

The pathological progression of lung cancer represents a multifaceted and dynamic process, encompassing stages from precancerous lesions to early-stage and late-stage cancer, as well as various treatment phases. Studies have demonstrated that the host’s continuously altering gut environment plays a important role in this progression [20,27]. Simultaneously, the patient’s gut microbiota undergoes continuous evolution. Notably, research has indicated that the gut microbiota exhibits significant parallel evolution alongside the lung cancer progression [28]. This raises important questions regarding whether this evolution follows a specific pattern and whether the temporal changes in the gut microbiota can offer insights that extend beyond traditional indicators for accurately staging, predicting therapy efficacy, and assessing prognosis in lung cancer. This topic will be explored in the following two sections. Furthermore, we have synthesized the variations in the composition and structure of gut microbiota across different stages of lung cancer and under various therapeutic modalities over the past five years (Table 1).

### 3.1. Dynamic Gut Microbiota in the Pathological Progression of Lung Cancer

#### 3.1.1. Dynamic Gut Microbiota in Early-Stage Lung Cancer

Initial investigations into benign lung nodules, often considered potential precursors or risk indicators for malignancy, have revealed a foundational disruption of the gut ecosystem. Specifically, patients with benign nodules exhibit a significant reduction in both microbial diversity and richness compared to healthy individuals [29].

As the pathological process progresses to early-stage lung cancer (ELC), the gut microbial landscape undergoes a more defined and potentially pro-carcinogenic shift. While certain core genera like *Bacteroides*, *Faecalibacterium*, and *Prevotella_9* may remain prevalent, their ecological context changes. Specifically, the community structure is reconfigured, marked by the decrease in typically beneficial genera like *Bifidobacterium* and *Roseburia* (as seen in healthy controls) and the increase in potential pathobionts such as *Klebsiella* and *Escherichia-Shigella* [30,31]. Actually, this alteration extends beyond a mere substitution of core taxa, encompassing significant disruptions in microbial interaction networks, functional pathways, and the overall ecological stability of the community. Furthermore, a key feature of ELC is the emergence and enrichment of distinct bacterial genera. For instance, *Mogibacterium* and *Ruminococcus* have been identified as specifically enriched in ELC patients [11,29].

This trend of dysbiosis intensifies across the broader lung cancer population. A consistent pattern observed in cancer cohorts is a marked compositional shift, defined by a disproportionate expansion of phyla like Bacteroidota and Proteobacteria, alongside a concurrent decline in Firmicutes and Actinobacteria. Furthermore, a decreased Firmicutes/Bacteroidetes ratio in the lung cancer cohort may lead to reduced levels of circulating SCFAs, thereby potentially affecting the host’s systemic immune response and inflammatory processes [11]. The translational potential of these microbial signatures is considerable. Researchers have successfully leveraged these specific gut microbiota operational taxonomic units (OTUs) to construct a non-invasive diagnostic tool—the patient discrimination index (PDI) [11]. This index, calculated as a weighted score from logistic regression models, demonstrates the feasibility of using gut microbiome profiles as sensitive and specific biomarkers for the early detection of lung cancer.

Synthesizing these findings, the gut microbiome in early-stage lung cancer is defined by several convergent features: (1) an overall loss of microbial diversity and stability; (2) a depletion of recognized beneficial microbiota (e.g., *Bifidobacterium*); and (3) a reciprocal enrichment of specific opportunistic pathogens or inflammatory taxa (e.g., *Klebsiella*, *Mogibacterium*). This characteristic “loss of protectors and rise of challengers” creates a systemic environment that may be permissive to tumorigenesis and immune evasion.

#### 3.1.2. Dynamic Gut Microbiota in Advanced Lung Cancer

In comparison to individuals with early-stage lung cancer and healthy controls, advanced lung cancer patients demonstrate a more pronounced and intricate dysbiosis of the gut microbiota. This dysbiosis is characterized not only by a general reduction in microbial diversity but also by a substantial reorganization of the microbial community structure, leading to the emergence of distinct microbial profiles associated with advanced disease states.

In patients with advanced lung cancer who have developed brain metastasis (BM), the gut microbiota exhibits similar characteristics to those observed in patients with early-stage lung cancer. Specifically, the relative abundance of potentially pathogenic bacterial phyla—such as Proteobacteria and Fusobacteria—increases, while the abundance of beneficial bacterial phyla that produce SCFAs and exert anti-inflammatory effects (e.g., Firmicutes and Actinobacteria) shows a decreasing trend. Based on these alterations, a biomarker panel consisting of *Faecalibacterium*, *Bifidobacterium*, *Butyricicoccus*, *Klebsiella*, *Streptococcus*, and *Blautia* has been developed and shown efficacy in differentiating patients with ELC and BM patients from healthy controls [30].

Furthermore, the gut microbiota in patients with advanced lung cancer demonstrates distinct characteristics, notably the enrichment of specific microbiota taxa that are not prevalent in the early stages of the disease. In patients with advanced lung cancer and brain metastasis, the gut microbiota is particularly enriched with taxa such as *Adlercreutzia*, *Caproiciproducens*, *Fournierella*, *Listeria*, and *Paenibacillus* [33]. The composition of these bacterial communities highlights microbiome features that are intricately linked to the progression of advanced lung cancer. This observation strongly indicates that the gut microbiota transcends being a mere passive reflection of the disease state and may actively contribute to shaping the microenvironment conducive to tumorigenesis.

The alterations in gut microbiota composition have substantial functional implications, as evidenced by changes observed in metabolomic profiles. In a mouse model of leptomeningeal metastasis (LM), significant disruptions were identified in the nicotinate/nicotinamide and tryptophan metabolic pathway [34]. Concurrently, metagenomic analyses of BM patients revealed modifications in the levels of immunomodulatory SCFAs and elevated concentrations of metabolites such as angiotensin [33]. These observations suggest that gut microbiota dysbiosis in advanced lung cancer extends beyond mere taxonomic shifts and involves extensive remodeling of the microbiota’s collective metabolic functions. Such metabolites may directly or indirectly influence the host’s immune status and metabolic pathways, thereby facilitating conditions conducive to tumor metastasis.

The distinctive characteristic of the gut microbiota composition in advanced lung cancer is its transition from general dysbiosis to a “specialized dysbiosis,” which is intricately linked to tumor progression. This transformation is not merely an intensification of the overgrowth of opportunistic pathogens observed in the early stages; rather, it involves the emergence of specific gut microbiota, such as *Listeria* and *Paenibacillus*, which are uncommon in the early stages. The proliferation of these genera indicates a significant remodeling of the gut microenvironment, creating conditions that facilitate tumor development. This observation suggests that the gut microbiota assumes a more active and specific role in advanced lung cancer compared to its role in the early stages; alterations in its composition are not solely a consequence of disease progression but are likely contributing factors that drive malignant behaviors.

Nonetheless, this analysis has limitations: due to the scarcity of studies on the gut microbiota composition in untreated advanced lung cancer patients without metastasis, our analysis is confined to patients with metastatic advanced lung cancer. More comprehensive and in-depth analyses will be needed in the future.

### 3.2. Dynamic Gut Microbiota During the Treatment Progression

#### 3.2.1. Radiotherapy

Radiotherapy, a critical therapeutic approach for lung cancer, demonstrates its effectiveness not only through the direct eradication of tumor cells but also through its significant systemic effects, notably on the gut microbial ecology. The gut microbiota is highly radiosensitive, and its composition plays a crucial role in determining the radiosensitivity of host cells. Studies have shown that the structure and function of the gut microbiota undergo significant remodeling during radiotherapy, and this change has a clear association with the efficacy of radiotherapy [42].

Radiotherapy has been observed to induce specific dynamic alterations in the gut microbiota. Studies have demonstrated that lung cancer patients who exhibit a favorable response to radiotherapy show an enrichment of particular microbial taxa within their gut microbiota, notably including *Blautia*, *Flavonifractor*, *Negativibacillus*, *Oscillibacter*, and *Prevotellaceae UCG-004*. Conversely, non-responders display a microbial composition dominated by Desulfovibrionaceae, *Prevotellaceae*, and *Megamonas* [35]. The majority of taxa in the former group are beneficial bacteria known for their production of SCFAs, whereas those in the latter group predominantly exert pro-inflammatory and pro-tumorigenic effects in various diseases. The distinct composition of gut microbiota between responders and non-responders strongly indicates that gut microbiota profiling could serve as a valuable tool for assessing the efficacy of radiotherapy.

Modulation of the gut microbiota represents a novel and promising strategy for enhancing the efficacy of cancer therapies. A study investigating the effects of oral vancomycin in conjunction with stereotactic body radiation therapy for lung cancer patients demonstrated that vancomycin administration leads to a reduction in microbial diversity. Specifically, it notably decreases the abundance of Bacteroidetes while increasing that of Proteobacteria, and significantly reduces the levels of SCFAs [43]. Notably, although this condition may initially seem to represent a state of dysbiosis, within the context of concurrent radiotherapy, this particular microbial imbalance can systematically activate dendritic cells and T cells. This activation enhances anti-tumor immunity, thereby ultimately augmenting the efficacy of radiotherapy.

Radiotherapy induces significant alterations in the gut microbiota composition of patients with lung cancer, and there exists a clear correlation between this altered microbiota structure and the therapeutic efficacy of radiotherapy. The distinct microbiota profiles observed between responders and non-responders imply that gut microbiota composition may serve as a predictive biomarker for radiotherapy outcomes. Importantly, under certain conditions, gut microbiota dysbiosis can paradoxically enhance the immune activation effects of radiotherapy. This suggests that the relationship between gut microbiota and the efficacy of radiotherapy is not merely a binary opposition of “balanced versus imbalanced”; rather, it is contingent upon the interaction mechanisms between specific microbiota structures and the host immune system. Consequently, precise modulation of the gut microbiota is anticipated to emerge as a novel strategy for optimizing radiotherapy approaches in the treatment of lung cancer. The central focus of this strategy is to cultivate a microbial environment that promotes anti-tumor immune responses, rather than solely aiming to maintain microbial diversity.

#### 3.2.2. Immunotherapy

Due to the limitations inherent in current detection methodologies and the absence of specific early diagnostic markers, a significant number of lung cancer patients are diagnosed at an advanced stage of the disease. For these patients with late-stage lung cancer, the effectiveness of conventional treatment modalities is frequently suboptimal. Consequently, early diagnosis and treatment of lung cancer are imperative for enhancing patient survival rates. In this context, immunotherapy presents a promising avenue in cancer treatment. Compared to traditional therapeutic approaches, immunotherapy offers greater specificity and durability, along with reduced side effects, thereby potentially enhancing patients’ quality of life. Among the various immunotherapeutic strategies, immune checkpoint inhibitors (ICIs) are the most prevalently employed in lung cancer treatment. ICIs function by augmenting the capacity of effector T cells to recognize and eradicate tumor cells through the targeting and modulation of critical inhibitory immune checkpoints, such as cytotoxic T-lymphocyte-associated protein 4 (CTLA-4) and PD-1, along with its ligand (PD-L1). Recent research suggests a significant correlation between the composition of gut microbiota and the effectiveness of ICI therapy [44,45]. The gut microbiota, as a vital ecosystem, is integral to the modulation of tumor immune responses, with its compositional and functional characteristics being closely associated with the success of immunotherapeutic interventions.

Extensive analyses have demonstrated that the gut microbiota composition in lung cancer patients undergoing immunotherapy exhibits distinct patterns correlated with clinical outcomes. Specifically, certain beneficial bacterial genera are consistently enriched in patients who respond favorably to immunotherapy. Numerous studies have shown that patients who exhibit effective responses to ICIs often possess gut microbiota abundant in genera capable of producing SCFAs, including *Faecalibacterium*, *Ruminococcus*, and *Akkermansia*. These microbial groups are thought to preserve the integrity of the intestinal barrier and enhance anti-tumor immunity through their metabolites. Conversely, an overabundance of bacterial genera such as *Desulfovibrio* and *Megamonas* is frequently observed in non-responders [37]. Another study examined the relationship between gut microbiota signatures and the outcomes of ICI therapy in lung cancer patients. The analysis demonstrated a significant enrichment of Archaea, specifically the phyla *Actinomycetota* and *Euryarchaeota*, in patients exhibiting short progression-free survival (PFS), while Bacillota was correlated with long PFS [38]. This distinct microbiota signature between “responders” and “non-responders” underscores the potential of the microbiome as a predictive biomarker.

The functions and metabolites of the gut microbiota play a crucial role in linking the gut microenvironment with systemic immunity. Metabolomic analyses have demonstrated that patients who respond to immunotherapy exhibit markedly different levels of immunomodulation-related metabolites. These include an enrichment of SCFAs, lysine, nicotinic acid, deoxycholic acid, and glycerol. Conversely, non-responders are characterized by elevated levels of metabolites such as 2-pentanone, tridecane, and L-citrulline. Importantly, a significant correlation has been observed between the gut microbiota and metabolite levels [39,46]. Therefore, the composition and dynamic alterations of the gut microbiota and metabolome may serve as valuable indicators for assessing the response to immunotherapy.

Research has shown a close correlation between the compositional characteristic of the gut microbiota and the clinical prognosis of lung cancer patients. In advanced NSCLC patients who received antibiotic treatment before ICIs therapy, their overall survival (OS) and PFS were significantly reduced (HRs of 2.55 and 2.52, respectively), and the objective response rate was also notably lower (25.9% vs. 55.6%) [47]. Moreover, a retrospective analysis of 118 patients with advanced NSCLC treated with immune checkpoint blockade found that after Clostridium butyricum therapy, patients had significantly prolonged PFS and OS, with HRs of 0.41 and 0.27, respectively [48]. These studies suggest that changes in the composition and structure of the gut microbiota may play a crucial role in influencing the survival rates of patients with lung cancer. Continuous monitoring and targeted interventions of the gut microbiota could enhance prognostic assessments and improve clinical outcomes for these patients.

Moreover, research has demonstrated that the gut microbiota can augment the effectiveness of immunotherapy by modulating specific metabolic pathways, notably those associated with amino acid, glycolysis, and bile acid metabolism [49]. Mechanistically, Bifidobacterium-derived extracellular vesicles are internalized by lung cancer cells through endocytosis, subsequently activating the TLR4-NF-κB signaling cascade to increase PD-L1 expression [50]. This discovery provides direct molecular evidence of the gut microbiota’s capacity to remotely influence the tumor immune microenvironment.

In conclusion, the composition of the gut microbiota and the levels of metabolites in lung cancer patients undergoing immunotherapy are intricately linked to the effectiveness of the treatment. The gut microbiota, through its distinct compositional traits, functional metabolic outputs, and complex interactions with the host immune system, plays a critical role in modulating the efficacy of lung cancer immunotherapy. The fundamental pattern of compositional changes is characterized by the dynamic equilibrium between “beneficial” and “detrimental” microbiota, while the functional pattern is manifested in the precise modulation of immune status via key metabolic pathways. Future research should prioritize a comprehensive analysis of the specific mechanisms through which these microorganisms and their metabolites exert their effects. Additionally, exploring strategies such as dietary interventions, probiotics, prebiotics, or fecal microbiota transplantation to modify the intestinal microenvironment may ultimately enhance the efficacy of immunotherapy and improve the prognosis of lung cancer patients.

#### 3.2.3. Chemoimmunotherapy

Chemoimmunotherapy has been established as a standard first-line or neoadjuvant treatment regimen for advanced lung cancer, significantly extending patient survival while maintaining manageable safety profiles. Nevertheless, patient responses to this therapy vary considerably. In recent years, research has progressively shifted its focus from the tumor itself to host-related factors. Among these, the gut microbiota has emerged as a critical component in regulating systemic immunity, playing increasingly prominent roles in predicting and modulating the efficacy of chemoimmunotherapy. Conducting an in-depth analysis of the alterations in the composition and function of the gut microecology under this treatment paradigm is of paramount importance for elucidating the mechanisms underlying variations in therapeutic efficacy, identifying potential biomarkers, and developing novel intervention strategies.

Patients who respond to chemoimmunotherapy exhibit notable commonalities in their gut microbiota composition. Primarily, increased microbial diversity and the presence of specific beneficial microbial taxa are distinguishing features of responders. A prospective study involving 106 patients with advanced NSCLC demonstrated that responders possessed significantly higher gut microbial α-diversity and distinct β-diversity. Their microbiota was characterized by an enrichment of Firmicutes and genera such as *Faecalibacterium* and *Subdoligranulum* [41]. Additionally, another cohort study identified several genera independently associated with prolonged OS, including *Bifidobacterium*, *Blautia*, *Butyricicoccus*, *Eubacterium ventriosum*, and *Fusicatenibacter*. Notably, most of these genera are recognized as SCFAs-producing bacteria, and the biosynthetic pathways for SCFAs exhibit increased activity in responders favorably, indicating potential functional synergy among them.

Patients who do not respond to treatment or have a poor prognosis demonstrate a contrasting state of dysbiosis within their gut microecology. Unlike the advantageous gut microbiota characteristics observed in responders, non-responders typically exhibit an gut environment enriched with taxa such as *Lactobacillus*, *Oscillibacter*, *the Prevotellaceae NK3B31 group*, and *Ruminococcaceae UBA1819*. Furthermore, some studies have also linked the overproliferation of *Bacteroides*, *Blautia*, and *Escherichia-Shigella* with treatment resistance [40,41].

In conclusion, within the realm of chemoimmunotherapy, the gut microbiota of lung cancer patients undergoes systematic alterations that are intricately linked to clinical outcomes. Responders favorably typically exhibit an gut microecology characterized by high microbial diversity, an abundance of the phylum Firmicutes, and the presence of specific SCFAs-producing microbiota, such as *Faecalibacterium*, *Bifidobacterium*, and *Blautia*. Conversely, non-responders tend to show an enrichment of particular genera, including *Lactobacillus* and *Oscillibacter*. The overarching pattern suggests that a healthy, balanced, and functionally active gut microenvironment—particularly one with a high capacity for SCFAs synthesis—appears to synergistically enhance the anti-tumor immune response elicited by chemoimmunotherapy. These findings underscore the potential of the gut microbiota as a prognostic biomarker and highlight a translational medicine approach aimed at improving clinical outcomes through microbiota modulation, such as fecal microbiota transplantation (FMT) and prebiotic/probiotic supplementation.

In summary, this chapter explores the evolving patterns of gut microbiota and their metabolites during lung cancer development, treatment, and prognosis. Unlike previous studies that view microbiota as static markers at single time points, this chapter advocates for dynamic monitoring to uncover temporal microbiome-host interactions, offering valuable clinical insights for monitoring disease progression, predicting treatment outcomes, and optimizing intervention timing. For instance, we emphasized the dynamic role of gut microbiota in lung cancer development and treatment, urging its redefinition as an active participant rather than a passive observer. This perspective aids in understanding its mechanisms, like immunomodulation and metabolite exposure, and supports the creation of personalized, stage-specific microecological therapies, such as microbial “preconditioning” before immunotherapy.

However, it is crucial to recognize that variations in technical platforms and analytical methods can cause inconsistent conclusions in studies. For instance, in gut microbiota research, 16S rRNA gene sequencing and metagenomic sequencing differ in species resolution and functional annotation [51]. Additionally, choosing different bioinformatics tools and reference databases affects species annotation and diversity analysis. This methodological variability complicates direct data comparisons and may result in inconsistent findings. Therefore, future studies should focus on: (1) standardizing methodologies, including sampling, storage, library construction, and analysis protocols for specific samples like feces and serum from lung cancer patients; (2) ensuring complete and transparent disclosure of methodological details in research reports to improve reproducibility and comparability; and (3) considering technical biases and adopting appropriate data integration and correction strategies in cross-study comparisons or meta-analyses. By unifying and optimizing methodologies, we can better understand the gut microbiota’s role in lung cancer and translate findings into clinically valuable biomarkers or intervention targets.

Notably, drugs and gut microbiota interact bidirectionally during treatment. Therapeutic interventions such as chemotherapy and antibiotics can disrupt microbial balance, while gut microbiota can influence the host’s response to these treatments [52,53]. This review examines predictive biomarkers and therapeutic targets by analyzing the differences in gut microbiota between responders and non-responders within the same treatment cohort, thereby mitigating the confounding effects of drug to some extent; however, certain limitations remain. Cross-sectional studies cannot determine if gut microbiota changes in lung cancer patients during treatment are a cause, effect, or co-evolution with the disease. Future research should focus on prospective studies to clarify these relationships and explore the potential of modifying gut microbiota as a treatment target, aiding in the clinical use of microbial biomarkers.

The gut microbiota’s composition and function are influenced by diet, geography, age, and comorbidities [54]. o minimize these confounding effects, we conducted longitudinal or cross-sectional comparisons within the same study, such as analyzing microbiota and metabolite differences between lung cancer patients and healthy individuals at the same time point, or within the same cohort before and after treatment. However, when synthesizing findings from multiple studies across different regions and populations, systematic biases from these factors can cause significant heterogeneity in identified biomarkers and their links to lung cancer, potentially leading to conflicting conclusions. Therefore, caution is warranted when interpreting cross-study conclusions, and the specific population context should be clearly defined. Future studies should use multicenter, prospective cohort studies to gather detailed metadata, including diet, geography, lifestyle, and clinical factors. Statistical methods like stratified analysis and multivariable adjustment can help control confounding factors, leading to a clearer understanding of the link between gut microbiota and lung cancer and its potential as a universal biomarker. However, residual confounding may still exist, so further validation through mechanistic studies or trials is necessary.

## 4. Promising Gut Microbiota and Metabolites

### 4.1. Promising Gut Microbiota

An increasing number of gut microbiota has been implicated in influencing the pathologic progression and immunotherapy of lung cancer. The gut microbiota plays a crucial role in disease development through regulating immune responses and their complex metabolic activities and interactions with the host. In this section, we will focus on several gut microbiota that have garnered significant attention in current studies. We summarize the differences in gut microbiota and metabolites in lung cancer diagnosis and immunotherapy in the last five years (Table 2). Given their potential roles in the progression and immunotherapy response of lung cancer, these gut microbiota are likely to become emerging strategies for the diagnosis and treatment.

#### 4.1.1. *Akkermansia muciniphila*

*A. muciniphila* is a Gram-negative bacterium belonging to the genus *Akkermansia* within the phylum Bacteroidetes. In 2004, *A. muciniphila* was first isolated from human fecal samples. *A. muciniphila* is primarily found in the intestines, particularly in the colon region. By utilizing mucus in the gut as a source of carbon and nitrogen, it helps maintain the integrity of the gut barrier. *A. muciniphila* can regulate the host’s metabolism and immune response, and also participate in the production of SCFAs (such as butyrate), which are crucial for gut health [60]. Some studies suggest that *A. muciniphila* may play a role in the prevention of certain diseases through reducing pathogen invasion by enhancing intestinal barrier function and alleviating inflammation by regulating immune responses [61].

Gut *A. muciniphila* may colonize lung cancer tissues through the bloodstream, thereby affecting lung cancer. *A. muciniphila* can regulate various metabolic pathways, including glutamine metabolism, purine and pyrimidine metabolism, and glycolysis. *A. muciniphila* exerts anti-cancer effects by regulating these metabolic pathways and reprogramming tumor metabolism [62]. Research shows that monocolonization with *A. muciniphila* or dual colonization with *A. muciniphila* and *E. hirae* reversed the compromised efficacy of PD-1 blockade observed after the recolonization of germ-free mice with FMT from non-responder patients. The restoration was IL-12-dependent, featuring an enhanced recruitment of CCR9+ CXCR3+ CD4 T lymphocytes into the tumor microenvironment of the mouse [63]. Moreover, metagenomic analysis of fecal samples from advanced NSCLC patients receiving PD-1 blockade therapy prospectively validated the prognostic significance of *A. muciniphila* in feces for advanced NSCLC patients undergoing ICI treatment. It was found that *A. muciniphila* is significantly associated with the prognosis of advanced NSCLC patients receiving ICI treatment [64]. A recent study has proposed an individual TOPOSCORE scoring system by exploring the relationship between the ecological topology of the gut microbiome and cancer immunotherapy, while also quantifying *Akkermansia* species. This system helps predict cancer patients’ responses to immunotherapy [65]. The rational application of *A. muciniphila* can provide new strategies and biomarkers for the diagnosis and treatment of lung cancer.

#### 4.1.2. *Prevotella copri*

As one of the primary species of the *Prevotella* genus. *P. copri* is widely distributed throughout the human body, particularly in the gastrointestinal and oral cavities. It is a Gram-negative, anaerobic bacteria that does not produce spores [66]. *P. copri* is associated with certain metabolic diseases and cancers, such as obesity, type 2 diabetes, lung cancer [24,67]. In the analysis of the gut microbiome of cancer patients, *P. copri* or its metabolites have been found to potentially affect the tumor microenvironment, thereby interfering with the body’s own anti-tumor immune response [68,69].

Research explored the impact of the human gut microbiome on cancer cachexia by combining shotgun metagenomics and plasma metabolomics in 31 lung cancer patients. The enrichment of BCAAs in non-cachectic patients was positively correlated with gut microbial species *P. copri*, suggesting a crucial role of *P. copri* and its metabolism in lung cancer [70]. In Chinese NSCLC patients receiving anti-PD-1 treatment, *P. copri*, *Alistipes putredinis*, and *Bifidobacterium longum* were observed to be enriched together among the responders, and patients with a beneficial gut microbiome, characterized by high diversity, showed improved memory CD8 T cell and natural killer cell profiles in their peripheral systems [20]. Mice transplanted with LC fecal microbiota or *P. copri* showed immunity functional disorder, while nervonic acid and all-trans-retinoic acid treatment enhanced inflammatory reaction and immunologic function in Lewis lung carcinoma (LLC)-bearing mice treated with *P. copri*. Furthermore, *P. copri* may be involved in the development of NSCLC by affecting the levels of sphingolipids and all-trans retinoic acid [24]. *Prevotella* may become a potential therapeutic target for intervening in the progression of NSCLC.

#### 4.1.3. *Veillonella parvula*

*Veillonella parvula* (*V. parvula*) is a strict Gram-negative anaerobic bacterium that is widely present in the human oral cavity, intestines, and respiratory tract. *V. parvula* has unique metabolic capabilities and plays an important role in various diseases, particularly in IBD and oral diseases [71]. In addition, its potential applications in cancer treatment have also garnered widespread attention.

*Veillonella* is enriched in patients with advanced lung cancer, prompting a poor prognosis. A study in *Kras*/p53 (KP) mice model found that *V. parvula* can induce lower respiratory tract inflammation and exacerbate tumor progression [72]. In patients with cancer cachexia due to lung cancer, the abundance of *Veillonella* is significantly increased. The abundance of *Veillonella* is positively correlated with the level of calprotectin, an inflammatory marker, in feces [73]. These findings open up new avenues for exploring the involvement of gut microbiota in cancer cachexia and its possible application as a therapeutic target.

*Veillonella dispar* is a branch of the *Veillonella* genus. The data indicate that the proportion of *Veillonella dispar* is higher in the high PD-L1 and immunotherapy responders group compared to the low PD-L1 and non-responders group [74]. This provided new insights for the development of gut microbiome-based biomarkers to predict immune therapy response.

### 4.2. Promising Metabolites

Lung cancer cells undergo “metabolic reprogramming” to support rapid growth, spread, and resistance to treatment, fundamentally altering their metabolic network. The most notable change is aerobic glycolysis, known as the Warburg effect. Furthermore, several critical metabolic alterations are evident, including those related to glutamine metabolism, lipid metabolism, and one-carbon metabolism [75]. The widespread metabolic dysregulation in lung cancer indicates that altered metabolism is a necessary factor for tumor initiation and progression, not just a byproduct of tumorigenesis. Notably, this systemic metabolic reprogramming does not occur in isolation; rather, it is significantly influenced by the interplay between the host and the environment, wherein the gut microbiota and its metabolites are increasingly acknowledged as key regulatory factors. The bidirectional regulation of the gut–lung axis is crucial for sustaining immune homeostasis and metabolic balance. Interaction between the gut microbiota and the lungs occurs through microbial components and metabolites that are transported to distant sites via the circulatory system. Microbial-derived metabolites (such as SCFAs, amino acids, neuroactive metabolites, etc.) can participate in lung cancer progression by regulating immune responses, inducing inflammation, releasing toxins, and secreting metabolites [28]. Dysbiosis may trigger metabolic disorders, leading to the release of pro-carcinogenic substances and the activation of carcinogenic signaling pathways that promote the development of lung cancer. In rare cases, gut microbiota can migrate to the lungs via the bloodstream, affecting the progression of lung diseases [76]. In this section, we mainly discuss how the metabolic products of the gut microbiota affect the pathological process of lung cancer.

#### 4.2.1. Short-Chain Fatty Acids

SCFAs, including butyrate, propionate, and acetate, are primarily produced by the fermentation of dietary fibers in the colon and cecum. They can be transported to distant organs via the circulatory system and are crucial for sustaining intestinal homeostasis and overall health, and preventing cancer [77]. Unmetabolized SCFAs in the gut enter the lungs via the peripheral circulation. There are small amounts of SCFAs in the lungs, and since there are no bacterial metabolic substrates in the lungs, the SCFAs found in the lungs are mainly produced by the gut microbiota [78,79]. A recent study has found that lung cancer patients, especially those with brain metastases, have a significant reduction in gut microbiota that produce SCFAs, with a marked decrease in fecal acetate and butyrate levels [30]. Another study found that there is dysbiosis of butyrate-producing bacteria in the gut of NSCLC patients: most butyrate-producing bacteria in the gut of NSCLC patients, including *Prevotella*, *Roseburia*, *Clostridium I*, *Ruminococcus*, *Clostridium XIVa*, and *Roseburia*, are notably reduced compared to healthy individuals [26]. Interestingly, butyrate-producing bacteria have been found to be abundantly expressed in the lung tissues of patients with recurrent lung cancer. Low concentrations of butyrate can stimulate the proliferation of lung cancer cells and increase the expression of H19 in tumor cells by inducing M2 macrophage polarization, which in turn facilitates the migration and invasion of lung cancer [80]. Another study found that after supplementing with probiotics, the number of microbiota producing SCFAs in the gut of mice increased, and higher levels of propionate and butyrate were also detected in the gut and blood, which promoted the expression of the chemokine CCL20 in pulmonary endothelial cells, thereby recruiting Th17 cells into the lungs, reducing the number of lung tumor lesions, and inhibiting the metastasis of tumor cells [81].

As a metabolite of significant interest in gut–lung axis research, the selection of appropriate biological samples is essential for the precise quantification of short-chain fatty acid levels. Current studies have focused primarily on detecting SCFAs in feces and plasma/serum, each of which has its own unique advantages and limitations [82]. SCFAs in feces accurately indicate local gut microbiota production but are difficult to analyze due to volatility during pre-treatment. While Serum/plasma SCFAs levels offer a direct indication of the metabolites involved in the gut–lung axis, their low concentrations demand highly sensitive and stable detection methods. Future studies should focus on optimizing cryopreservation and extraction protocols for fecal samples to reduce pre-analytical losses and enhancing analysis techniques for detecting trace metabolites.

#### 4.2.2. Neuroactive Substances

The gut microbiota can secret neurotransmitter precursors, which can be directly catalyzed into neurotransmitters through metabolism, or indirectly mediate the production and secretion of neurotransmitters by enteroendocrine cells through their metabolites. It has been found that the gut microbiota can modulate the release of various neurotransmitters, such as glutamate, Gamma-aminobutyric acid (GABA), dopamine, and 5-hydroxytryptamine (5-HT) [83]. These neurotransmitters transmit signals to various tissues through the vagus nerve or the blood and lymphatic systems to exert their effects. Interestingly, more and more neurotransmitters are being found to play regulatory roles in lung cancer. In this section, we will mainly introduce three neuroactive substances that play roles in lung cancer.

GABA is an inhibitory neurotransmitter that can be converted through glutamate decarboxylase, participating in various metabolic and physiological activities. Several gut bacterial genera have been recognized for their significant roles in the regulation of GABA production. For example, *Bacteroides* and *Lactobacillus* in the gut can regulate glutamate decarboxylase to metabolize glutamate into GABA [84]. Furthermore, GABA prevents macrophage polarization to the M1 type by inhibiting the NF-κB pathway and STAT3 pathway, while promoting macrophage M2 polarization by activating the STAT6 pathway. Moreover, GABA could increase the expression of FGF2 in macrophages and thus promote tumor neovascularization [85].

Serotonin (also known as 5-HT) is a biogenic amine and neurotransmitter synthesized from tryptophan, which is involved in many physiological processes including maintaining gastrointestinal function, vasoconstriction, and immune modulation [86]. Most of the 5-HT in the human body is synthesized from tryptophan absorbed from food by enterochromaffin cells (EC) in the intestinal epithelium. Early studies found that spore-forming bacteria in the gut can enhance the secretion of 5-HT by ECs and affect their normal function in the body [87]. However, the 5-HT metabolic pathway has been shown to promote tumorigenesis. Higher levels of 5-HT were found in the tumor tissues of NSCLC patients, potentially facilitating the onset and progression of NSCLC via the 5-HT/c-Myc/SLC6A4 signaling pathway. This tumorigenic effect can be effectively inhibited by 5-HT inhibitors [88]. There was an overexpression of the 5-HT7 receptor (HTR7), one of the receptors for 5-HT, in NSCLC tumor tissues as opposed to the nearby normal lung tissues. NSCLC patients with high HTR7 expression levels showed a connection to lymph node metastasis and advanced TNM stages. Moreover, HTR7 may promote migration and invasion of NSCLC cells by activating P38 or Src signaling pathways [89].

Dopamine (DA) is a catecholamine neurotransmitter produced primarily by dopaminergic neurons in the brain. However, DA is also synthesized in the gut, particularly in enterochromaffin cells and some neurons in the intestine. Gut microbiota such as *Lactobacillus* sp. and *Bifidobacterium* can convert tyrosine to DA or L-DOPA via tyrosine decarboxylases, and L-DOPA is rapidly converted to DA by dopa decarboxylase in the small intestine due to its instability [90]. There are five receptors for DA, of which the most frequently studied in lung cancer are dopamine receptor D2 (DRD2) and dopamine receptor D2 (DRD1). DA signaling significantly affects tumor growth and immune regulation through DRD in lung cancer. Compared to adjacent normal lung tissues, DRD2 is expressed less in NSCLC tissues, and its overexpression reduces NSCLC cell viability and growth by inhibiting the NF-κB signaling pathway [91]. Another receptor, DRD1, was also found to be downregulated and have increased methylation levels in lung cancer. DRD1 inhibits tumor cell proliferation by suppressing the activation of the EGFR/MAPAK/ERK signaling pathway axis, suggesting that the loss of DRD1 expression and methylation are predictive markers for lung cancer progression [92].

Based on these observations, lung cancer appears to disrupt the homeostasis of the intestinal microbiota, leading to dysbiosis that modifies neurotransmitter release and consequently impairs the microbiota’s regulatory influence on tumor behavior. These disturbances may create a self-perpetuating cycle that further accelerates malignant progression. Consequently, precisely targeting the interactions among gut microbes, their neurotransmitter metabolites, and lung cancer could provide a novel and promising strategy for the diagnosis and treatment of this malignancy.

#### 4.2.3. Other Metabolites

The gut microbiota can directly or indirectly secret some amino acids, such as lysine, histidine, sulfur-containing amino acids, and tryptophan and its derivatives. Tryptophan is an aromatic essential amino acid. In addition to being metabolized into 5-HT by enterochromaffin cells, the gut microbiota can also break down tryptophan into indole and its derivatives [83]. Significantly lower levels of tryptophan and kynurenine were detected in NSCLC patients, while levels of 3-hydroxyanthranilic acid (3-HAA) were significantly higher than healthy volunteers. After ICI treatment, both tryptophan and 3-HAA levels were decreased, and patients with lower 3-HAA levels had better efficacy and longer PFS. These results suggest that the detection of tryptophan and its derivatives may help predict the efficacy of ICI treatment in NSCLC patients [93]. In an association analysis based on gut microbiomics and metabolomics conducted on lung cancer patients, it was found that L-valine was reduced in lung cancer patients, and the abundance of *Lachnospiraceae_UCG-006*, which is most strongly correlated with L-valine, also decreased in lung cancer patients [94].

The gut microbiota has been found to influence the metabolism of lipids in the host’s blood and tissues [95]. In the metabolomics analysis of early-stage NSCLC patients, higher levels of sphingolipids, fatty acyls, and glycerophospholipids were detected, and their metabolism may be regulated by *Clostridium* and *Muribaculaceae* [31]. Another similar study also found high levels of fatty acyls and glycerophospholipids in the serum metabolites of lung cancer patients [56]. Although the specific mechanisms by which lipid metabolism affects lung cancer development are not yet clear, these results suggest that lipid metabolism regulated by the gut microbiota is likely to influence the prognosis and treatment of lung cancer.

Bile acid (BA) derivatives produced through gut microbiota modulation can regulate intestinal diseases and tumorigenesis. Higher levels of deoxycholic acid (DCA), ursodeoxycholic acid (UDCA), and chenodeoxycholic acid (CDCA) were observed in the serum of NSCLC patients compared with healthy control groups. Additionally, patients with elevated DCA levels show increased expression of G protein-coupled receptor 5 (TGR5), with a strong positive correlation between them. DCA promotes the migration and invasion of NSCLC cells in a TGR5-dependent way [96]. A study based on serum untargeted metabolomics conducted a metabolic analysis on NSCLC patients and found that the levels of glycocholic acid (GCA), cholic acid (CA), and glycoursodeoxycholic acid (GUDCA) were elevated in NSCLC patients [97]. In another study, the serum levels of primary BAs were generally elevated in NSCLC patients, while the levels of secondary BAs such as lithocholic acid (LCA) were decreased [98]. These differentially expressed BAs and their derivatives are very likely to associate with the development of lung cancer, though more specific mechanisms need further research.

**Figure 2:**
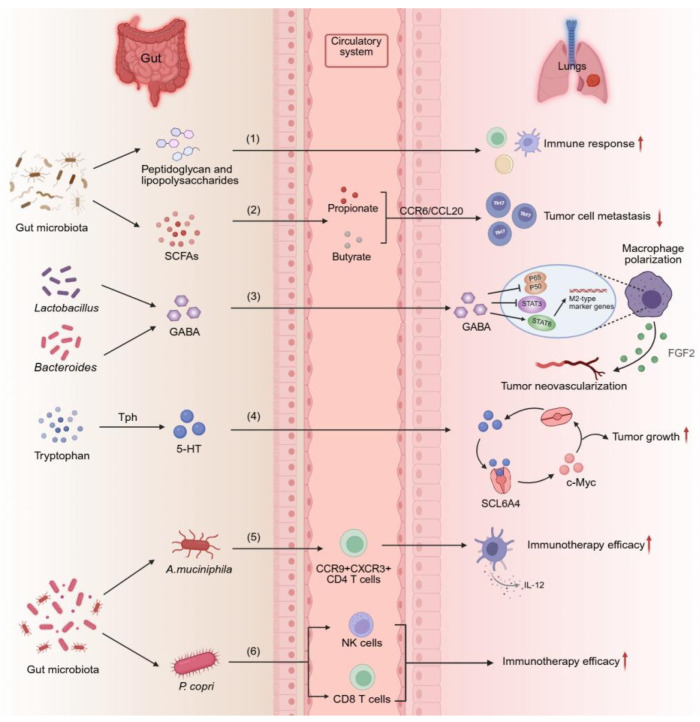
The mechanism by which gut microbiota and metabolites affect the immune microenvironment in lung cancer. (1) Peptidoglycan and lipopolysaccharide directly stimulate lung immune responses via the circulatory system. (2) Microbial-derived SCFAs recruit Th17 cells in the lung tumor microenvironment through the CCR6/CCL20 axis, inhibiting tumor cell metastasis. (3) Bacteroides and Lactobacillus promote the production of GABA, GABA inhibits the NF-κB and STAT3 pathways while activates the STAT6 pathway to induce M2 polarization of macrophages, and promotes tumor neovascularization via the FGF2 pathway. (4) 5-HT promotes the growth of tumor cells through the 5-HT/c-Myc/SLC6A4 signaling loop. (5) A.muciniphila recruit CCR9+ CXCR3+ CD4+ T lymphocytes and induce dendritic cells to secrete IL-12, thereby enhancing the efficacy of PD-1 blockade treatment. (6) *P. copri* enhances immune efficacy by stimulating memory CD8 T cells and natural killer cells. Arrow definition: ↑ indicates increase/enhancement; ↓ indicates decrease/suppression. Created in BioRender. Xiao, X. (2026) https://BioRender.com/re9f21j.

Based on these studies, we conclude that certain gut microbiota and metabolites that participate in regulating immunity to a large extent, and have complicated regulatory effects on the lungs (Figure 2). The specific mechanisms by which the gut microbiota and its metabolites affect lung cancer are still unclear, and research on gut microbiomics and metabolomics in lung cancer diagnosis and treatment is still in its early stages. However, there is a substantial correlation between the gut microbiota and its metabolites and lung cancer initiation and progression, which may affect the treatment response in lung cancer patients. The combined analysis of gut microbiome and metabolomics helps identify biomarkers related to the occurrence and treatment response of lung cancer, providing a basis for early diagnosis and personalized treatment.

## 5. Future Perspectives

Research on gut microbiota is an interdisciplinary field combining microbiology, genetics, bioinformatics, and ecology to study the structure, function, and changes in gut microbial communities. It holds promise for cancer diagnostics and therapy. The “gut–lung axis” has highlighted the crucial role of gut microbiota in lung cancer development, progression, and treatment response. This concept reveals how gut microbiota and their metabolites influence lung cancer by affecting the immune system and local inflammation, linking gut dynamics with lung tumor processes. We have compiled the dynamic changes in gut microbiota related to lung cancer progression and treatment response. These variations highlight the biological traits of different disease stages and offer valuable insights for precision medicine, from early diagnosis to treatment enhancement.

While this review provides an initial exploration of the patterns and characteristics of gut microbiota in the progression of lung cancer and response to treatment, it is important to acknowledge that significant challenges remain in translating these findings into clinical practice. At present, there is a lack of large-scale and prospective clinical validation, and the precise molecular mechanisms underlying these observations require further elucidation. In addition, the precise dynamic monitoring and quantification of gut microbiota and metabolites, which play critical roles in the gut–lung axis, are contingent upon the advancement of systematic pretreatment protocols and the optimization of analytical methodologies. For example, fecal samples with volatile SCFAs need immediate cryopreservation and rapid transport to prevent losses. Gas chromatography–mass spectrometry is preferred for its high resolution and sensitivity in quantifying SCFAs [99].

Most studies on the gut microbiota and lung cancer currently rely on cross-sectional analyses, which failed to capture the dynamic changes during disease progression and treatment. This limits our understanding of the dynamic gut–lung axis. Future research should focus on three aspects: (1) using integrated multi-omics analyses (metagenomics, metabolomics, immunomics, etc.) to identify key microbial species, metabolic pathways, and immune biomarkers involved in lung cancer, and create a comprehensive “microbiota-metabolism-immunity” network; (2) encouraging clinical cohort studies to collect biological samples at various stages of lung cancer treatment and develop microbiota-focused trials, such as combining probiotics or dietary interventions with immunotherapy, to assess the impact and benefits of microbiota modulation in humans; and (3) combining longitudinal clinical sampling (tracking the full-course dynamics of microbiota from precancerous lesions to advanced lung cancer) with fecal microbiota transplantation experiments in humanized mouse models to explore how microbiota influences lung cancer progression, facilitating the translation from clinical observation to mechanistic understanding. The primary objective is to advance the gut microbiota into a personally modifiable diagnostic biomarker and therapeutic target, incorporating it into comprehensive guidelines for the holistic management of lung cancer.

Consequently, developing a robust model for lung cancer diagnosis and treatment efficacy prediction based on dynamic gut microbiota is a complex and demanding endeavor. Future research is essential to advance the “dynamic microbiota”-based diagnostic and therapeutic model towards the realization of precision medicine.

## Figures and Tables

**Table 1:** Gut microbiota composition across different tumor progression and therapeutic regimens of lung cancer.

Species	Tumor Progression/Therapeutic Regimens	Gut Microbiota	Enrichment Cohort	Ref
Human	Early-stage lung cancer	Clostridiales, Selenomonadales, *Mogibacterium*	LC	[29]
		*Bacteroidales*, *Acetivibrio*, *Sutterella*, *Eisenbergiella*	Controls	
Human	Early-stage lung cancer	*Bacteroides*, *Faecalibacterium*, *Klebsiella*, *Phascolarctobacterium*, *Prevotella_9*	LC	[30]
		*Bacteroides*, *Faecalibacterium*, *Phascolarctobacterium*, *Prevotella_9*, *Roseburia*	Controls	
Human	Early-stage lung cancer	*Bacteroides*, *Escherichia-Shigella*	LC	[31]
		*Bifidobacterium*, *Dialister*, *Megamonas*, *Prevotella_9*	Controls	
Human	Early-stage lung cancer	*Ruminococcus*	LC	[11]
		*Bifidobacterium*, *Faecalibacterium*, *Streptococcus*, *Veillonella*	Controls	
Human	Early-stage lung cancer	*Apiotrichum*, *Aspergillus*, *Saccharomyces*	LC	[32]
		*Candida*	Controls	
Human	Advanced lung cancer	*Bacteroides*, *Escherichia/Shigella*, *Faecalibacterium*, *Klebsiella*, *Prevotella_9*	LC	[30]
		*Bacteroides*, *Faecalibacterium*, *Phascolarctobacterium*, *Prevotella_9*, *Roseburia*	Controls	
Human	Advanced lung cancer	*Adlercreutzia*, *Caproiciproducens*, *Fournierella*, *Listeria*, *Paenibacillus*	brain metastasis	[33]
		Fusobacteria, *Parabacteroides distasonis*	without distant metastasis	
Mice	Advanced lung cancer	*Alistipes*, *Lactobacillus*, *Ligullacoccus*	leptomeningeal metastasis	[34]
		Bacteroidaceae, Lachnospiraceae, Rikenellaceae, *Alistipes*, *Bacteroides*, *Muribaculum*	Controls	
Human	Radiotherapy	*Blautia*, *Flavonifractor*, *Negativibacillus*, *Oscillibacter*, *Prevotellaceae UCG-004*	responsive	[35]
		Desulfovibrionaceae, Prevotellaceae, *Megamonas*	non-responsive	
Human	CCRT	Firmicutes	Short PFS	[36]
		Bacteroidota, Proteobacteria	Long PFS	
Human	ICIs	*Desulfovibrio*, *Megamonas*	non-responders	[37]
		*Akkermansia*, *Blautia*, *Faecalibacterium*, *Ruminococcus*	responders	
Human	ICIs	*Actinomycetota*, *Euryarchaeota*	Short PFS	[38]
		Bacillota	Long PFS	
Human	ICIs	*Brachybacterium* sp. SGAir0954, *Burkholderia anthina*, *Candidatus Thioglobus* sp. NP1, *Nocardioides* sp. dk3136	non-responders	[39]
		*Bradyrhizobium guangdongense*, *Corynebacterium stationis*, *Methanococcus vannielii*, *Plantactinospora* sp. BC1	responders	
Human	ICIs	*A. tamarii*	non-responders	[12]
		*C. bacterium*, *CG. bacterium* MH-37, virus crAssphage cr127-1	responders	
Human	Chemoimmunotherapy	*Lactobacillus*, *Oscillibacter*, *Prevotellaceae NK3B31 group*, *Ruminococcaceae UBA1819*	non-responders	[40]
		*Bifidobacterium*, *Blautia*, *Butyricicoccus*, *Eubacterium ventriosum*, *Fusicatenibacter*	responders	
Human	Chemoimmunotherapy	*Bacteroides*, *Blautia*, *Escherichia-Shigella*	non-responders	[41]
		Firmicutes, *Faecalibacterium*, *Subdoligranulum*	responders	

**Table 2:** Profiles of gut microbiota and metabolites in lung cancer patients.

Species	Cohort	Sample Type	Gut Microbiota	Metabolites and Metabolic Pathways	Enrichment Cohort	Ref
Human	LC vs. controls	Feces	Proteobacteria, *Bacteroides*, *Ruminococcus*	Steroid biosynthesis, bile secretion	LC	[11]
			Firmicutes, Actinobacteria, *Faecalibacterium*, *Streptococcus*, *Bifidobacterium*, *Veillonella*, *Bacteroides*	Flavonol biosynthesis apoptosis, G protein-coupled receptors	Controls	
Human	LC vs. controls	Feces	*Granulicatella*	dodecane, 2,6-dimethyl-4 heptanone, methyl isobutyl ketone	LC	[55]
			Rikenellaceae, Peptostreptococcaceae, Mogibacteriaceae, Clostridiaceae, Prevotellaceae, *Akkermansia muciniphila*, *Bacteroides caccae*	SCFAs, aldehydes, ketones, terpenes andp-cresol	Controls	
Human	LC vs. controls	Feces, Serum	Halanaerobiaeota, *Actinomyces*, *Veillonella*, *Megasphaera*, *Enterococcus*, *Clostridioides*	Fatty Acyls, Glycerophospholipids, PC, prenol lipids	LC	[56]
			Tenericutes, Cyanobacteria	Imidazopyrimidines	Controls	
Human	LC tumor tissues vs. normal tissues	Tissues	Actinobacteria, *Mmethyloversatilis discipulorum*	Histone H2A, histone H3	LC tumor tissues	[57]
			Proteobacteria, *Enterococcus faecium*, *Helicobacter pylori*	SETD8	Normal tissues	
Human	LC vs. controls	Feces	*Limosilactobacillus gorillae*, *Streptococcus salivarius*,	-	LC	[58]
			*Akkermansia muciniphila*, *Alistipes shahii*, *Prevotella copri*, *Phocaeicola coprophilus*	SCFAs, tryptophan, GABA, histamine, secondary bile acid metabolism	Controls	
Mice	LC vs. controls	Feces	Lachnospiraceae, *Acutalibacter*	Acetate synthesis I, acetate degradation, propionate synthesis III, butyrate synthesis I, GABA synthesis III	LC	[58]
			Firmicutes, Lachnospiraceae, Muribaculaceae, *Akkermansia muciniphila*	Tryptophan degradation, melatonin synthesis, secondary bile acid metabolism	Controls	
Mice	LC vs. controls	Feces	Lachnospiraceae	C4-dicarboxylic acid cycle, reductive pentose phosphate cycle, amino acid metabolism, UMP, IMP biosynthesis	LC	[59]
			*Ligilactobacillus murinus*, *Bacteroides acidifaciens*	Resistance, ribonucleotide biosynthesis, lipopolysaccharide, lipid biosynthesis	Controls	
Human	Responders vs. non-responders	Feces, Serum	*Butyricicoccus*, *Adlercreutzia*, *Allisonella*	D-ribose, flavin adenine dinucleotide, 3-amino-4-hydroxybenzoate, methionine sulfoximine	Responders	[45]
			*Klebsiella*, *Erysipelatoclostridium*	Glutarylcarnitine, 4-acetamidobutanoic acid, maltotriose, purine, and PAGln	Non-responders	
Human	Responders vs. non-responders	Feces	*Faecalibacterium prausnitzii*, *Blautia faecis*, *Anaerobutyrium hallii*, *Eubacterium ramulus*, *Coprococcus catus*, *Akkermansia muciniphila*, *Bifidobacterium bifidum*	SCFAs, Acetyl-CoA pathway	Responders	[44]
			*Enterococcus faecalis*, *Prevotella copri*	Propionic acid consumption pathway	Non-responders	

## Data Availability

No new data were created or analyzed in this study.

## Abbreviations

The following abbreviations are used in this manuscript:

55-HT	5-hydroxytryptamine
*A. muciniphila*	*Akkermansia muciniphila*
BA	Bile acid
BM	brain metastasis
CA	Cholic acid
CCL20	C-C Motif Chemokine Ligand 20
CDCA	Chenodeoxycholic acid
CTLA-4	Cytotoxic T-lymphocyte-associated protein 4
DA	Dopamine
DCA	Deoxycholic acid
DRD1	Dopamine receptor d1
DRD2	Dopamine receptor d2
EC	Enterochromaffin cells
ELC	Early-stage lung cancer
FMT	Fecal microbiota transplantation
GABA	Gamma-aminobutyric acid
GCA	Glycocholic acid
GUDCA	Glycoursodeoxycholic acid
ICIs	Immune checkpoint inhibitors
KP	*Kras*/p53
LCA	Lithocholic acid
LLC	Lewis lung carcinoma
LM	Leptomeningeal metastasis
NK cells	natural killer cells
NSCLC	Non-small cell lung cancer
OS	Overall survival
OTU	Operational taxonomic unit
*P. copri*	*Prevotella copri*
PD-1	Programmed cell death protein 1
PD-L1	Programmed cell death-ligand 1
PDI	Patient discrimination index
PFS	Progression free survival
SCFAs	Short-chain fatty acids
TGR5	G protein-coupled receptor 5
Th17 cells	T helper 17 cells
UDCA	Ursodeoxycholic acid
*V. parvula*	*Veillonella parvula*
